# Supplementary material for: CTRP13-Mediated Effects on Endothelial Cell Function and Their Potential Role in Obesity
Source: Cells. 2024 Jul 31;13(15):1291. doi: 10.3390/cells13151291 (PMC11311976; doi:10.3390/cells13151291)
Supplement: Supplementary file 1 [file cells-13-01291-s001.zip › cells-2989194-supplementary.pdf]

**CTRP13-Mediated Effects on Endothelial Cell Function and Their Potential Role in Obesity**

Muhammad Aslam [1,#], Ling Li [2,#], Sina Nürnberger [2], Bernd Niemann [3]
and Susanne Rohrbach [2]

[1] Department of Cardiology and Angiology, [2] Institute of Physiology and [3]Department of Cardiac and Vascular Surgery, Justus Liebig University Giessen

[#] Both authors contributed equally.

Corresponding author:
Susanne Rohrbach
Institute for Physiology
Justus Liebig University Giessen
Aulweg 129
35392 Giessen
Germany
susanne.rohrbach@physiologie.med.uni-giessen.de
Phone: 0049-641-9947268
Fax: 0049-641-9947269

**Supplement**

**Supplementary Table S1: Primer sequences**

| | **GenBank accession #** | **Forward Primer** | **Reverse Primer** |
|---|---|---|---|
| CTRP13 human | NM_001010908.2 | GCA ATT GCC CAA GAT GCT GAT | TTT CCT CCATGG GCT TTC CC |
| PRKAA1 human | NM_006251.6 | GCG CAG ACT CAG TTC CTG | TCT TCA CAG CTA CTT TAT GCC C |
| PRKAA2 human | NM_006252.4 | AAG ATC GGA CAC TAC GTGC | ATT TTT CCA ACA ACA TCT AAA CTGC |
| TNF-alpha human | NM_000594.3 | CCC ATG TTG TAG CAA ACC CTC | TAT CTC TCA GCT CCA CGC CA |
| ICAM-1 human | NM_000201.3 | TGT GAC CAG CCC AAG TTG TT | TGG AGT CCA GTA CAC GGT GA |
| IL-8 human | NM_000584.4 | GCT CTG TGT GAA GGT GCA GTT | ACC CAG TTT TCC TTG GGG TC |
| Col1A1 human | NM_000088.4 | CCT GGT GAA TCT GGA CGT GA | ACC CAC GAT CAC CAC TCT TG |
| Col3A1 human | NM_000090.4 | ACG GAA ACA CTG GTG GAC AG | GAA GCT CGG CTG GAG AGA AG |
| alpha-SMA human | NM_001613.4 | GAG TCT GCT GGC ATC CAT GA | GTA CAT AGT GGT GCC CCC TG |
| TGF-beta1 human | NM_000660.7 | CGT CTG CTG AGG CTC AAG TT | CTA AGG CGA AAG CCC TCA AT |
| GAPDH human | NM_002046.7 | GTC ACC AGG GCT GCT TTT AAC | TTG ACG GTG CCA TGG AAT TTG |
| HPRT-1 human | NM_000194.3 | TGA CCA GTC AAC AGG GGA CA | TGC CTG ACC AAG GAA AGC AAA |
| 18S rRNA human | X03205.1 | TGG AGC GAT TTG TCT GGT TA | ACG CCA CTT GTC CCT CTA AG |
| CTRP13 rat | NM_001109403 | AGA GCC CGG ACC | GCG ATC TTG GGC ACC GTG |

| | | | |
|---|---|---|---|
| | | ACC GGG | CT |
| 18S rRNA rat | NR_046237 | TGG AGC GAT TTG TCT GGT TA | ACG CCA CTT GTC CCT CTA AG |
| GAPDH rat | NM_017008.4 | CAT CAC CAT CTT CCA GGA GGG | GGT TCA CAC CCA TGA CGA ACA |
| HPRT-1 rat | NM_012583.2 | CGA GCC GAC CGG TTC TGT CAT G | GAG GGC CAC AAT GTG ATG GCC TC |
| CTRP13 mouse | NM_153155.3 | GGC AAG TTC ACC TGC TCC AT | GAG CAA TTG CAC TAG CAC GC |
| GAPDH mouse | BC023196.2 | CAT CAC CAT CTT CCA GGA GCG | CGT TTG GCT CCA CCC TTC AA |
| HPRT-1 mouse | NM_013556.2 | GAT CAG TCA ACG GGG GAC AT | AGA GGT CCT TTT CAC CAG CAA |
| 18S rRNA mouse | NR_003278.3 | TGG AGC GAT TTG TCT GGT TA | ACG CCA CTT GTC CCT CTA AG |

**Supplementary Figures**

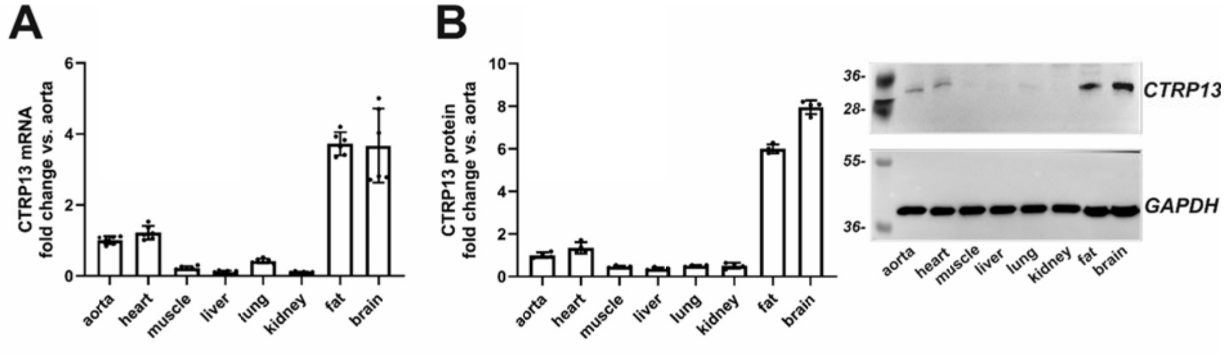


**Supplementary Fig. S1: CTRP13 mRNA and protein expression in various mouse organs**
(**A**) Results from qPCR analyses of CTRP13 mRNA expression in various organs of wild-type mice. (**B**) Quantification of CTRP13 protein expression in various mouse organs (left panel) and representative CTRP13 western blots (uncropped images with size markers as indicated) from these organs (right panel). GAPDH served as the loading control. All data represent mean ± SEM from 6 male animals.

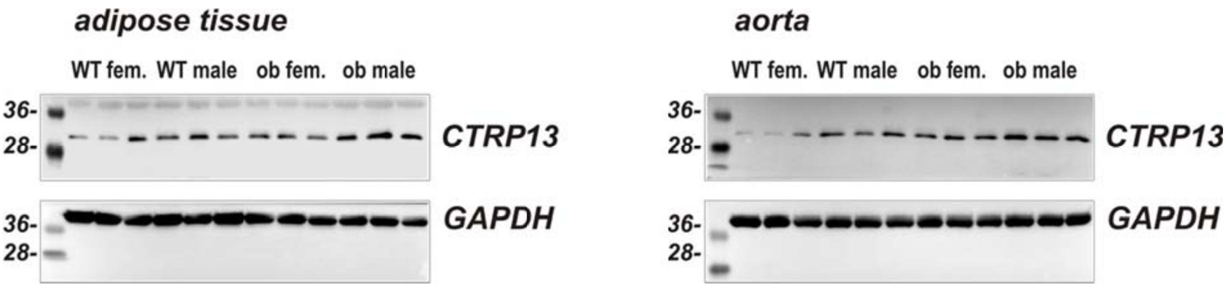


**Supplementary Fig. S2: CTRP13 expression in adipose and aortic tissue of obese and lean mice**
Representative CTRP13 western blots (uncropped images with size markers as indicated) from adipose tissue (left panel) and aortic tissue (right panel) of male and female wild-type or ob/ob mice. GAPDH served as loading control.

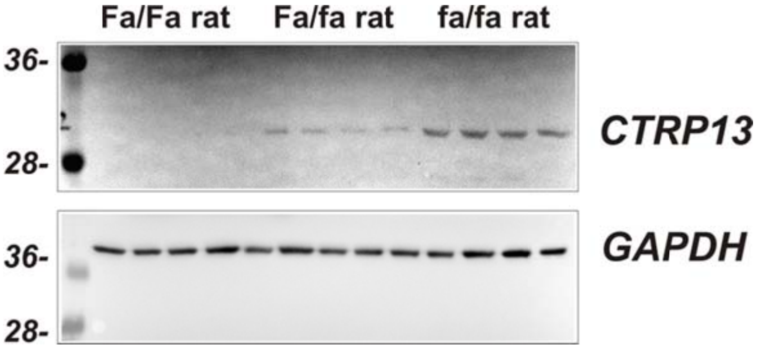


**Supplementary Fig. S3: CTRP13 expression in the aorta of ZDF rats**
Representative CTRP13 western blots (uncropped images with size markers as indicated) from aortic tissue of male obese ZDF (fa/fa) rats, lean heterozygous (Fa/fa) rats and wild-type (Fa/Fa) rats. GAPDH served as loading control.

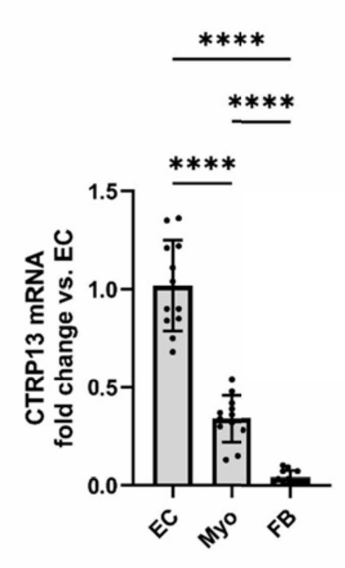


**Supplementary Fig. S4: CTRP13 mRNA expression in rat cardiac endothelial cells, cardiomyocytes or cardiac fibroblasts**
CTRP13 mRNA expression was analyzed in adult rat cardiac endothelial cells (EC), cardiomyocytes (Myo) or cardiac fibroblasts (FB) by qPCR. Results are presented relative to CTRP13 expression in ECs. Data are shown as mean±SEM, with n=12 per group. ****: $p<0.0001$.

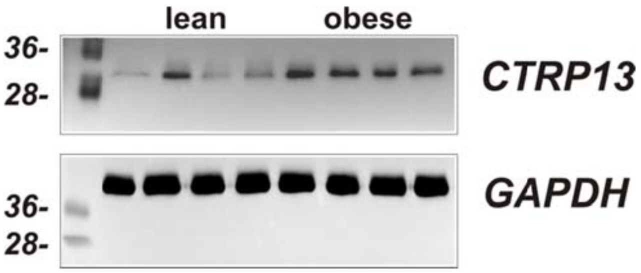


**Supplementary Fig. S5: CTRP13 expression in the mammary artery of obese and lean patients**

Representative CTRP13 western blots (uncropped images with size markers as indicated) from mammary arteries of lean (BMI 18.5-25 kg/m$^2$) or obese (30-35 kg/m$^2$) patients. GAPDH served as loading control.

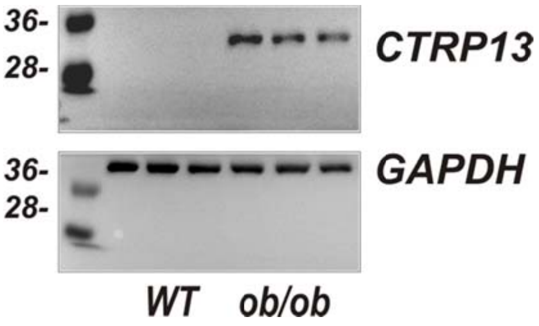


**Supplementary Fig. S6: Induction of CTRP13 in HUVECs by mouse serum**

Representative CTRP13 western blots (uncropped images with size markers as indicated) from cells grown in Opti-MEM™ supplemented with either 10% serum from wild-type (WT) or ob/ob mice for 48h. GAPDH served as loading control.

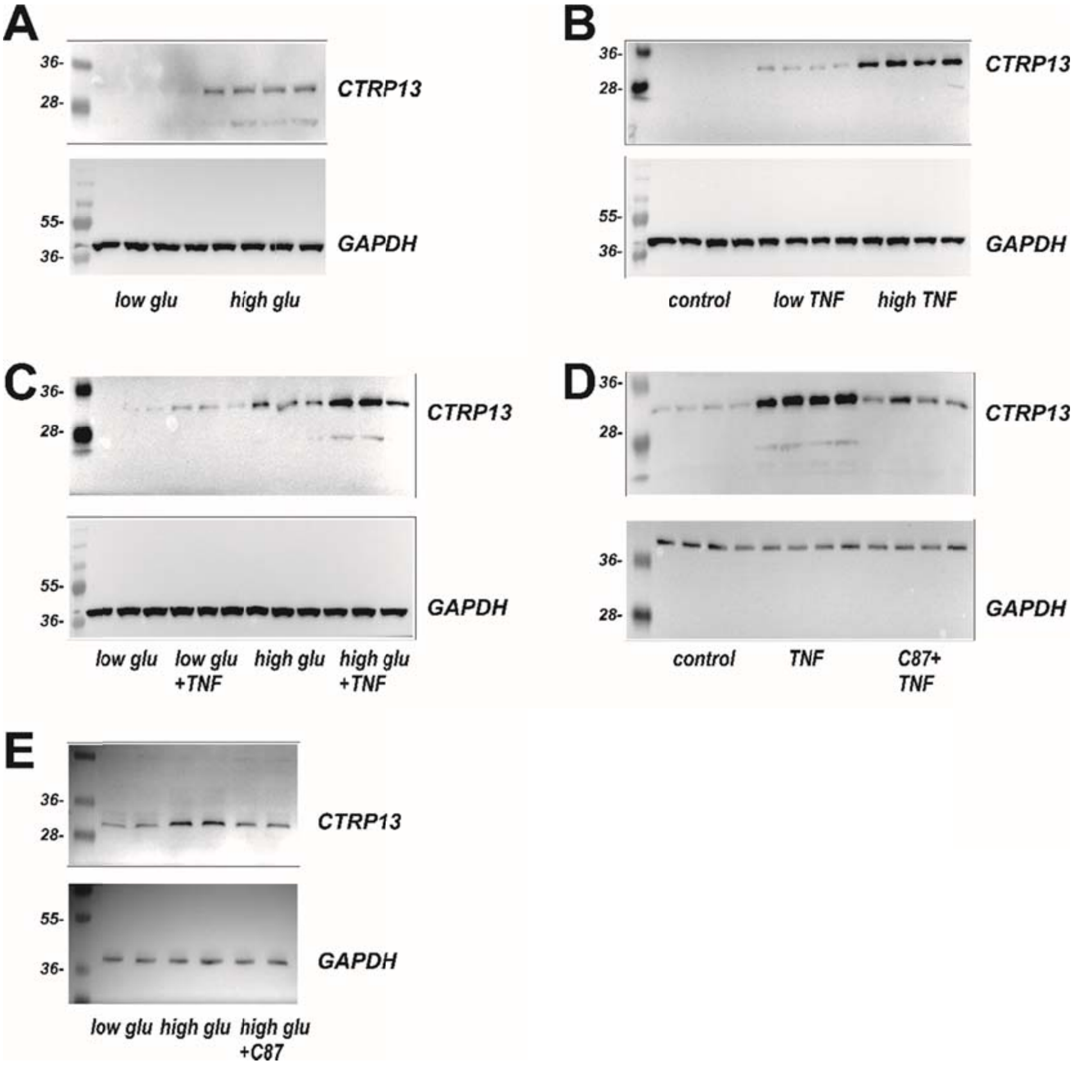


**Supplementary Fig. S7: Impact of high glucose and TNF-alpha on CTRP13 protein expression in HUVECs**

Representative CTRP13 western blots (uncropped images with size markers as indicated) from (**A**) HUVECs cultured under normal glucose conditions (5 mM D-glucose, low glu) or high glucose conditions (25 mM D-glucose, high glu) for 24 h. (**B**) HUVECs cultured under normal glucose conditions (5 mM D-glucose, low glu) or treated with 1 ng/ml or 10 ng/ml TNF-alpha for 24 h. (**C**) HUVECs cultured under normal glucose conditions (5 mM D-glucose, low glu) or high glucose conditions (25 mM D-glucose, high glu) and treated ± 1 ng/ml TNF-alpha for 24 h. (**D**) HUVECs cultured under normal glucose conditions (5 mM D-glucose, low glu), treated with ± 1 ng/ml TNF-alpha for 24 h, and preincubated with the TNF-alpha-specific small-molecule inhibitor C87 (2 µM) for 60 min as indicated. GAPDH served as loading control.

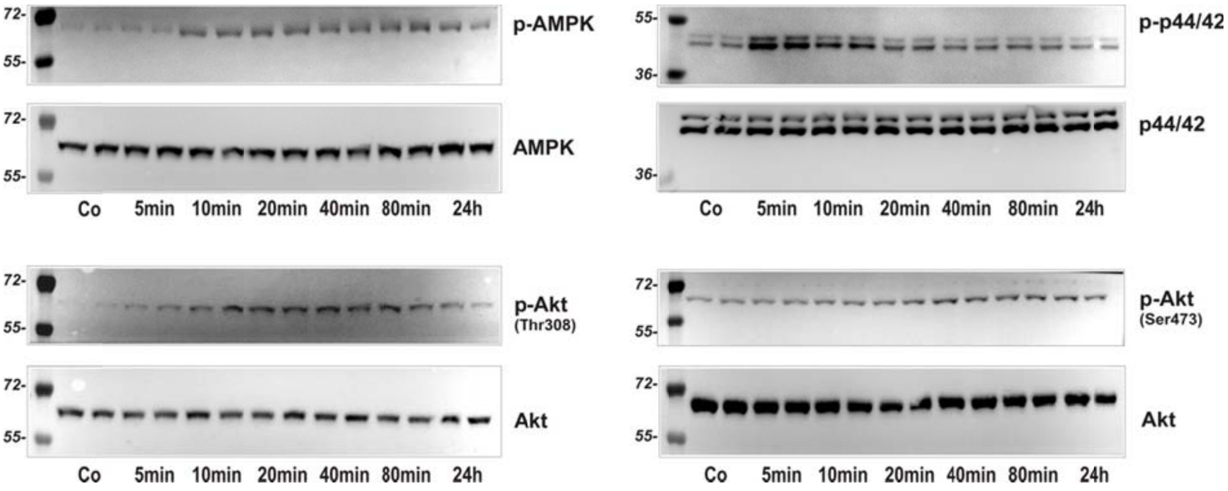


**Supplementary Fig. S8: Time course of signaling pathway activation in response to CTRP13 in HUVECs**

Representative western blots (uncropped images with size markers as indicated) for phosphorylation of AMPK (Thr172), p44/42 MAPK (Thr202/Tyr204) and Akt (Thr308 or Ser473) after treatment with CTRP13 (4 µg/ml) for the indicated time in Opti-MEM™ supplemented with 1% FBS. Total AMPK, total p44/42 MAPK or total Akt served as loading control.

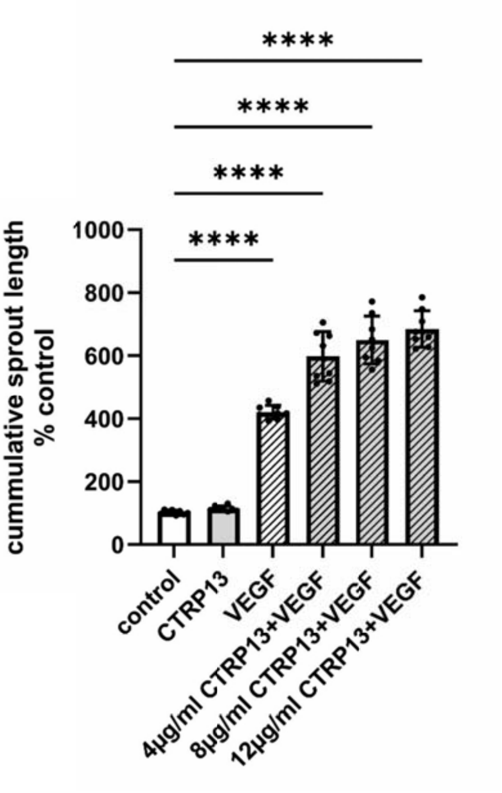


**Supplementary Fig. S9: Sprouting assay**

Cumulative sprout length. HUVECs were cultured as hanging drops to form spheroids. The spheroids were embedded into a collagen matrix and incubated with EGM containing 4 µg/ml CTRP13, 25 ng/ml VEGF or VEGF + CTRP13 at increasing concentration (4, 8, 12 µg/ml), and then incubated at 37°C overnight in a cell culture incubator. After 24 h, cell sprouts were photographed and cumulative sprout length was analyzed. Data are mean ± SEM from 4 independent experiments with 2 biological replicates each. ****p< 0.0001 vs. control.

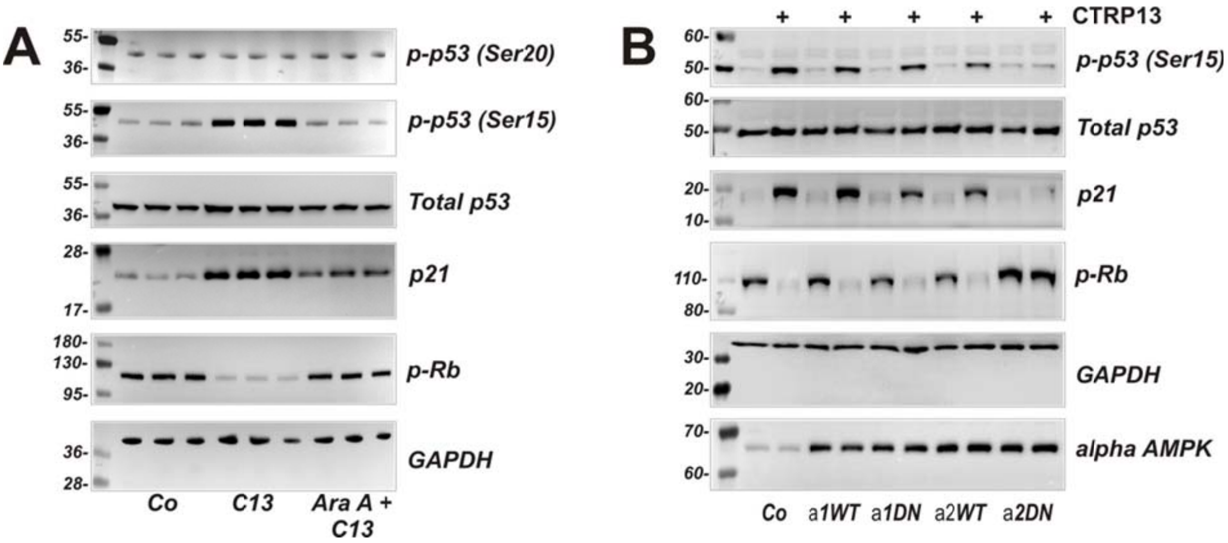


**Supplementary Fig. S10: Impact of CTRP13 on cell cycle regulators and cell cycle progression**

(**A**) Cells were either left untreated in Opti-MEM™ supplemented with 1% serum or treated with CTRP13 (4 µg/ml) for 24 h. Sixty minutes prior to CTRP13 treatment, HUVECs were incubated with the AMPK inhibitor AraA (500 µM) as indicated. Representative western blots (uncropped images with size markers as indicated) for phosphorylation of p53 (Ser15 or Ser20) or Rb and protein expression of p53 and p21. GAPDH served as loading control. (**B**) Adenoviral overexpression of wild-type (WT) or dominant negative (DN) alpha 1 and alpha 2 AMPK was performed in HUVECs. Forty-eight hours later cells were either left untreated in Opti-MEM™ supplemented with 1% serum or treated with CTRP13 (4 µg/ml) for 24 h. Western blots were performed as described in (A).

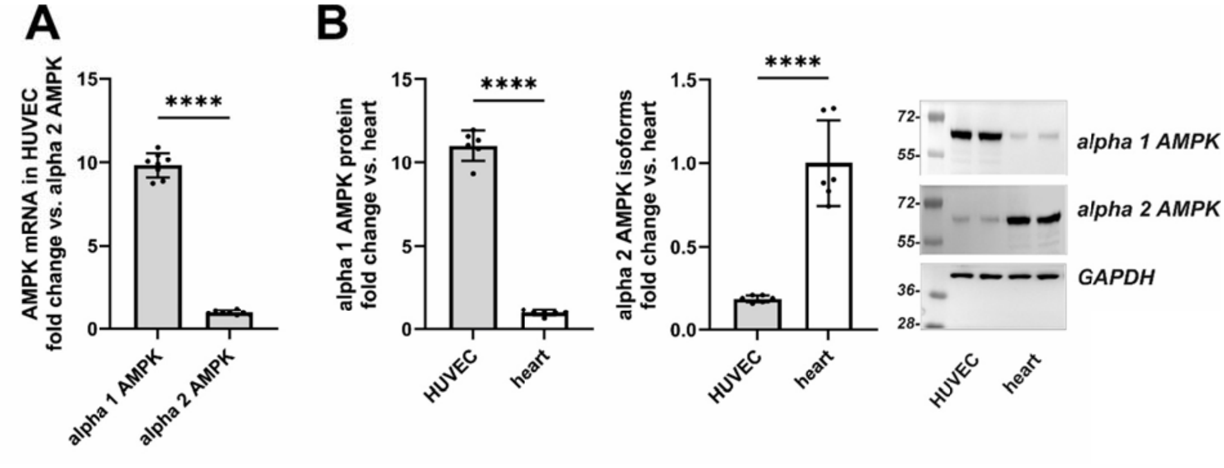


**Supplementary Fig. S11: alpha AMPK isoform expression in HUVECs**

(**A**) Alpha 1 and alpha 2 AMPK mRNA expression was analyzed in HUVECs by qPCR. (**B**) Quantification of CTRP13 protein expression in HUVECs (left panel). Results are presented in comparison to alpha 1 and alpha 2 AMPK expression in human cardiac tissue. Representative western blots (uncropped images with size markers as indicated) from HUVECs and human cardiac tissue (right panel). GAPDH served as loading control. Data are shown as mean±SEM, with n=6-8 per group. ***: p<0.001.

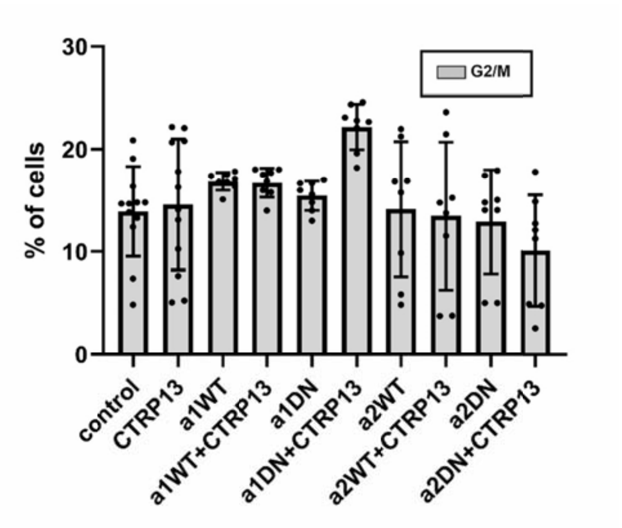


**Supplementary Fig. S12: Role of alpha AMPK isoforms in mediating the CTRP13 effects on cell cycle progression (G2/M phase)**

Adenoviral overexpression of wild-type (WT) or dominant negative (DN) alpha 1 and alpha 2 AMPK was performed in HUVECs. Forty-eight hours later cells were either left untreated in Opti-MEM™ supplemented with 1% serum or treated with CTRP13 (4 µg/ml) for 24 h. Results from FACS-based cell cycle analyses. Only the results regarding the impact of alpha AMPK isoforms in mediating the CTRP13 effects on the G2/M phase are depicted. Data are shown as mean ± SEM from 4 independent experiments with 2-3 biological replicates each.

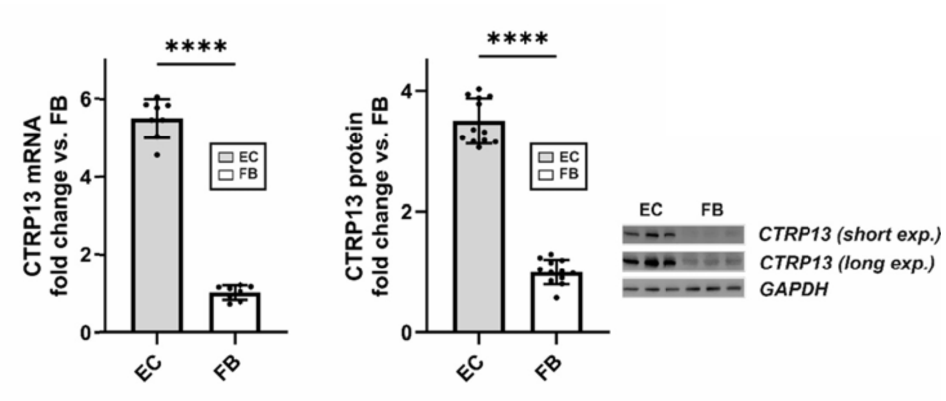


**Supplementary Fig. S13: CTRP13 expression in human endothelial cells and human fibroblasts**

(**A**) CTRP13 mRNA expression was analyzed in HUVECs and human RA fibroblasts by qPCR. (**B**) Quantification of CTRP13 protein expression in HUVECs and human fibroblasts (left panel). Representative western blots (uncropped images with size markers as indicated) from HUVECs and human fibroblasts (right panel). GAPDH served as loading control. Data are shown as mean ± SEM, with n=8-12 per group. ****: p<0.001.

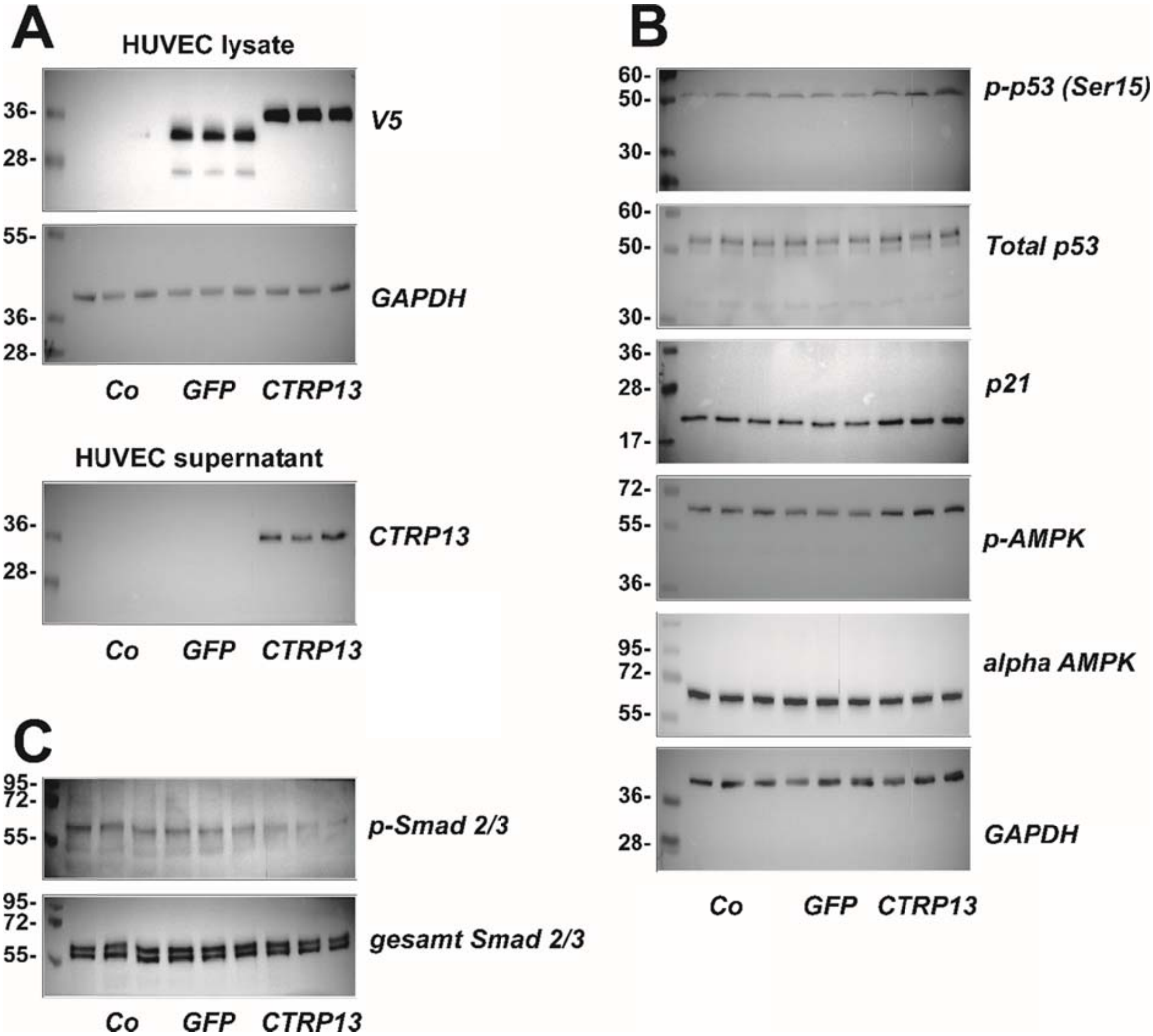


**Supplementary Fig. S14**: **Impact of endothelial CTRP13 on human fibroblasts.**
Adenoviral overexpression of CTRP13 or GFP in HUVECs was performed. Conditioned medium from HUVECs overexpressing either CTRP13 or GFP or HUVECs treated with conditioned medium from 293A cells without overexpression was utilized to culture human fibroblasts in 50% (vol/vol) HUVECs medium in DMEM for 24 h. (**A**) Overexpression of V5-tagged CTRP13 or GFP in HUVEC lysates (upper panel) or secretion of CTRP13 into cell culture medium (lower panel) was analyzed by Western blotting. GAPDH served a loading control. (**B**) Western blots were performed for phosphorylation of p53 (Ser15), total protein expression of p53, p21 and alpha-AMPK and AMPK phosphorylation (Thr172). GAPDH served as loading control. (**C**) Western blots were performed for phosphorylation of Smad2 (Ser465/467)/ Smad3 (Ser423/425).
